# Herring roe oil exerts immunomodulatory effects on the IL-17/23 signaling axis in immune and skin cells as models for psoriasis

**DOI:** 10.3389/fmed.2026.1684328

**Published:** 2026-08-24

**Authors:** Jennifer Mildenberger, Nina Therese Solberg, Tone-Kari K. Østbye, Vibeke Høst, Federico Petruccelli, Runhild Gammelsaeter, Sissel B. Rønning, Mona E. Pedersen

**Affiliations:** 1 Møreforsking AS, Ålesund, Norway; 2 Nofima AS, Ås, Norway; 3 Arctic Bioscience AS, Ørsta, Norway

**Keywords:** cell proliferation, inflammation, n-3 pufa, phospholipids, psoriasis, skin abnormalities

## Abstract

Several systemic treatment options are available for moderate to severe psoriasis, while low-risk oral treatments for milder forms are limited. Herring roe oil has previously shown beneficial effects on the clinical presentation of psoriasis, however, cell-specific effects remained unknown. This study aimed to elucidate cellular effects of herring roe oil and whether signaling pathways in skin and immune cells relevant to the pathophysiology of psoriasis are affected by herring roe oil. We used cell-culture models of human primary macrophages and T-cells, a keratinocyte cell line and human primary fibroblasts to represent important site-specific pathological aspects of psoriasis in the skin and immune system. The cells were supplemented with herring roe oil emulsions and cellular signaling was analyzed on protein and mRNA level. Skin cell proliferation was analyzed in real-time by a Live-Cell Analysis System. We here present results which suggest that herring roe oil exerts effects on different cell types on major psoriasis-driving aspects, reducing the secretion of IL-23 and IL-17 from macrophages and T-cells, respectively. Herring roe oil also interfered with IL-17-induced expression of the psoriasis marker *S100A7* and limited the proliferation of keratinocytes in co-culture with fibroblasts. Our results on cellular effects of herring roe oil demonstrate relevance in immunomodulating pathways known to be of importance in psoriatic pathophysiology and are in line with the potential clinical use of herring roe oil in the treatment of psoriasis.

## 1 Introduction

Psoriasis is a chronic immune-mediated inflammatory disease (IMID) affecting the skin. The exact etiology is unknown but involves genetic and environmental influences. The physical manifestations of psoriasis are characterized by hyperproliferation and abnormal differentiation of keratinocytes, leading to scaly plaques, and by a strong inflammatory component manifesting in massive infiltration of immune cells (1, 2). It is recognized that the regulation of skin immune functions happens in the epithelial immune microenvironment between mainly keratinocytes in the epidermis and immune cells and fibroblasts in the papillary dermis (3–5).

In the development of psoriasis, keratinocytes are triggered by external stimuli to release cytokines and antimicrobial peptides (AMPs). These form a complex with nucleic acids and are sensed by plasmacytoid dendritic cells (pDC) and macrophages, leading to the secretion of Interleukin 1-alpha (IL-1α) and Tumor necrosis factor alpha (TNF-α), further stimulating the maturation of resident dermal DCs and infiltrating monocyte-derived inflammatory DCs. Mature and activated DCs secrete cytokines such as IL-12, IL-23, Interferon gamma (IFN-γ) and TNF-α and present antigens to naïve T-cells, which differentiate into Th1, Th17 and Th22 helper cells. Th1 and Th17 cells secrete mainly IFN-γ and IL-17, respectively (2). The secretion of Th17 cytokines promotes keratinocyte hyperproliferation and cytokine secretion, again stimulating T-cells and attracting DCs, neutrophils, and macrophages (6). Despite much focus on DCs, macrophages are involved in both initiation and sustained inflammation in psoriasis. They shift to a pro-inflammatory polarization characterized by secretion of IL-23, TNF-α and IL-1β, as well as neutrophil-attracting chemokines, all with a driving role in psoriasis (3). Psoriatic patients have increased numbers of circulatory monocytes and in mouse models, depletion of macrophages has ameliorated clinical signs of psoriasis (7, 8).

Both keratinocytes and fibroblasts are affected by and contribute to the aberrant inflammatory signaling in the psoriatic skin. Keratinocytes express IL-17 receptor and binding of IL-17 activates the transcription factor nuclear factor kappa-light-chain-enhancer of activated B cells (NF-κB) and mitogen-activated protein kinases (6). The NF-κB inhibitor zeta (NFKBIZ, encoding IκBζ protein) has recently been shown to be a positive regulator of NF-κB and central mediator of IL-17-driven effects (9, 10). IκBζ in keratinocytes is further involved in the upregulation of psoriasis-associated genes, such as the AMP and psoriasis marker S100A7 (also known as psoriasin) (11, 12). S100A7 has been linked to impaired differentiation of keratinocytes and overall amplified inflammation (13). Fibroblasts are also targets of IL-17 and capable of sustaining inflammation by the production of proinflammatory mediators and growth factors (14, 15). Notably, fibroblasts are one of the first cell types to respond to blockage of IL-23 (16). In addition to keratinocytes and fibroblasts, other non-immune cells in the dermis, such as mesenchymal stem cells play pivotal roles in regulating inflammatory conditions in the skin (5).

In moderate-to-severe psoriasis, several biologics with effect on the IL-17/IL-23 axis have shown successful treatment outcomes in recent years, interfering with the vicious cycle of over-activation (1, 17), but there have been few treatment advances for patients with mild-to-moderate disease, where the use of biologics is inappropriate. Most psoriasis patients (80%) have non-severe disease (18), and their current treatment options are mostly limited to topical immunosuppressive drugs such as corticosteroids, and phototherapy. Current treatment options are dissatisfactory for patients with mild disease in terms of efficacy, ease-of-use and safety profile. There is an unmet medical need for safe, cost-efficient and oral treatment options for mild-to-moderate psoriasis patients.

Omega-3 polyunsaturated fatty acids (n-3 PUFAs) have established immunomodulatory effects, which are relevant to the amelioration of psoriasis (19, 20). They are further metabolized to specialized pro-resolving mediators (SPMs) with therapeutic potential in IMIDs (19, 21). Nevertheless, there is no consensus on the anti-psoriatic effects of n-3 PUFAs in clinical trials (22). This might be due to differences in the composition and chemical structure of the studied lipids (23–26), what makes it interesting to further investigate promising formulations in more detail. Herring (*Clupea harengus*) roe oil (HRO) has a high content of marine n-3 PUFAs, of which 66% are docosahexaenoic acid (DHA), 22% eicosapentaenoic acid (EPA) and 3% docosapentaenoic acid (DPA). These fatty acids are to an important part (35%) bound as phospholipid esters. HRO has previously been shown to improve the clinical manifestation in patients with mild *Psoriasis vulgaris* (27, 28), which was supported by immunomodulatory effects (29). While clinical studies are gold standard for drug development, cell models can offer molecular insights, which might not be visible at the systemic level, and which can be important to understand the overall picture. We have previously shown that supplementation of HRO in macrophages and keratinocytes promotes the biosynthesis of SPMs by these cells (30). Here, we characterized effects of HRO on cellular responses of immune and skin cells that are relevant in the context of psoriasis to elucidate the mode-of-action of HRO on a molecular level.

## 2 Materials and methods

### 2.1 Herring roe oil

Herring (*Clupea harengus*) roe was used for extraction of HRO, and cells were supplemented as previously described (30). HRO contains a high amount of phospholipid esters (35%) enriched with n-3 PUFAs, what allows the creation of emulsions of HRO in water. A 5% (w/w) emulsion of HRO in dH_2_O was prepared by stirring at 800 rpm under inert atmosphere (nitrogen flushing) at 37 °C for 1 h. Emulsions were then pasteurized under inert atmosphere by sonication at 70 °C for 2 min in an ultrasonic water bath (Branson 2200, 40 kHz) or by an ultrasonic probe. Emulsions were stored at 4 °C for maximum 4 days prior to cellular experiments. For treatment of cells, the prepared emulsions were further diluted in cell medium to 5–500 μg/mL and dH_2_O was used as control. The highest used concentration of HRO of 500 μg/mL provides slightly less n-3 PUFAs than previously measured in the plasma of healthy volunteers after supplementation with HRO (31).

### 2.2 Cell culture

#### 2.2.1 Immune cells

Buffy coats were obtained from the blood bank Ålesund (Helse Møre og Romsdal, Norway) with approval by the Regional Ethics Committee (REK, ref.: 230804). General exclusion criteria for blood giving applied and donors have given informed consent for the use of their blood in this study. Peripheral blood mononuclear cells (PBMC) were isolated by LymphoprepTM (AXIS-SHIELD PoC AS,1114547) density gradient and monocytes were selected by plastic adherence and differentiated into monocyte-derived macrophages (MDM) by culture in AIM V medium (Gibco, 12055083) with 5% CTS™ Immune Cell Serum Replacement (ICSR) and 10 ng/mL M-Csf (Miltenyi Biotec, 130-093-963) for 5 d, followed by incubation without M-Csf. To induce inflammatory signaling, cells were treated with 1 μg/mL lipopolysaccharide (LPS, Sigma-Aldrich, L2630) and 50 ng/mL IFN-γ (Miltenyi Biotec, 130-096-481). These concentrations are within a commonly used range (32–34) and were selected to provide a robust induction of IL-23. MDMs were pre-treated with HRO to allow for its cellular uptake before addition of inflammatory stimuli and to exclude physical interference of HRO in the cell medium with inflammatory stimuli.

For T-cell isolation, PBMC were stored overnight in AIM V with 5% ICSR and CD^4+^ T-cells were isolated by negative magnetic bead separation using the CD^4+^ T Cell Isolation Kit (Miltenyi Biotec, 130-096-533). T-cell purity was checked by staining for CD4 (Miltenyi, 130-113-790), CD3 (Miltenyi, 130-113-700) and CD14 (Miltenyi, 130-110-577) and analysis on a BD Accuri Flow cytometer (NTNU, Ålesund) with visualization in FCSalyzer 0.9.22-alpha and was routinely above 95% (Supplementary Figure 1). T-cells were activated by CD3/CD28 Dynabeads™, at 1 bead/2 cells (Gibco, 11161D) and cytokines [IL-1β (20 ng/mL, Invitrogen, A42508), PGE_2_ (5 μM, Cayman chemicals, 14010), IL-23 (25 ng/mL, Miltenyi Biotec, 130-095-757)] for 3 d. T-cells were co-treated with HRO during activation as a longer process, and not to delay activation by pre-treatment. For co-culture of MDMs with autologous T-cells, T-cells were either cultured with 1 ng/mL IL-7 or were frozen until differentiation of MDMs. MDMs were pretreated with HRO for 16 h or not, followed by activation with LPS for 24 h. T-cells were either pretreated with HRO for 16 h and added in fresh medium 1:1 on the activated MDMs for 3 d, or the co-culture was co-treated with HRO.

THP1-Dual™ NF-κB-SEAP IRF-Luc Reporter monocytes (InvivoGen, thpd-nfis) were cultured in RPMI 1640 (Thermofisher, 61870036) with 10% heat-inactivated fetal bovine serum (FBS) (Sigma-Aldrich, F0804), 100 U/mL penicillin + 0.1 mg/mL streptomycin (Sigma-Aldrich, P4333). The cells were differentiated to MDMs by seeding with 40 ng/mL Phorbol 12-myristate 13-acetate (PMA; Sigma-Aldrich, P8139) and incubation for 3 d, followed by 1.5 d in fresh medium without PMA. Cells were pre-treated with HRO and stimulated with 10 ng/mL LPS to induce inflammatory signaling. All immune cells were cultured in 5% CO_2_ at 37 °C.

#### 2.2.2 Skin cells

Human primary dermal fibroblasts (ATCC PCS-201-012) and human keratinocytes HaCaT (CLS Cell line service GmbH, cat no 300493) were cultured in 5% CO_2_ at 37 °C in Dulbecco’s modified Eagle’s medium (DMEM) high glucose supplemented with 10% FBS, 100 U/mL penicillin and 100 μg/mL streptomycin (all from Thermo Fisher Scientific) before seeding at passages 4–6. Two different co-culture systems were used, with the Transwell^®^ system chosen for analysis of the response of HRO on keratinocytes, and with fibroblasts as co-regulators. This system allows for separate isolation of each cell type for end-point analysis. A physical co-culture system was used to monitor a general effect of HRO on skin cells.

For the co-culture experiments in Transwell^®^ (TW) 12-well plates (Corning Costar Transwell, VWR), 1 × 10^5^ fibroblasts were seeded at the well bottom and cultured for 2 d before 5.5 × 10^5^ keratinocytes were seeded on the TW filters and co-cultured for 5 d before stimulation. Stimulation was performed for 4 d (to allow for the establishment of cell-cell communication between cell types) with or without 100 ng/mL IL-17A (Sigma-Aldrich, cat.no SRP0675) and with or without 5 or 50 μg/mL HRO, before sampling of media and cells.

For direct co-culture, 3 separately grown populations of fibroblasts were plated as 3.6 × 10^3^ or 9.86 × 10^3^ cells per well in respectively 12-well plates for RNA and 6-well plates for protein. After 2 d of cultivation, 3 separately grown populations of keratinocytes were added on fibroblast containing plates at 1.5 × 10^4^ cells/well in 12-well plates, and 4.11 × 10^4^ cells/well in 6-well plates. Cells were co-cultured for 5 d before stimulation for 4 d as in the TW system. For proliferation studies, 2.0 × 10^3^ fibroblasts or 4.0 × 10^3^ keratinocytes were plated per well in a 96-well plate for monoculture monitoring. For co-culture monitoring, 500 fibroblasts were mixed with 2.5 × 10^3^ keratinocytes in a 96-well plate. Stimuli were added to the cells 1 d after plating, and real-time cell confluence was subsequently monitored every 4 h using an IncuCyte^®^ S3 Live-Cell Analysis System (Sartorius AG, Germany).

### 2.3 Cellular viability

Effects on cellular viability of the immune cells were analyzed by a resazurin-based assay (PrestoBlueTM; Thermofisher, A13261) and spectrophotometric detection on a Synergy HTX S1LFA plate reader (BioTek) according to the manufacturer’s instructions. The viability of fibroblasts and HaCat was evaluated using CellTiter-Glo^®^ Luminescent Cell Viability Assay (Promega) following the manufacturer’s protocol. Viability percentages were calculated relative to untreated control cells (100% viability).

### 2.4 Reporter assays

NF-kB or IRF activation were analyzed according to the manufacturer’s instructions (35) by secreted embryonic alkaline phosphatase (SEAP) (QUANTI-Blue™, InvivoGen, rep-qbs) or Lucia luciferase (QUANTI-Luc™, InvivoGen, rep-qlc1) reporter assay in undiluted cell supernatants from THP1 reporter cells after stimulation with 10 ng/mL LPS for 24 h. QUANTI-Luc™ was analyzed immediately by luminescence and QUANTI-Blue™ after 30–60 min of incubation by absorbance at 635 nm on a Synergy HTX S1LFA plate reader (BioTek).

### 2.5 Enzyme-linked immunosorbent assay (ELISA) and BioPlex

Cytokine secretions were analyzed in duplicates in appropriately diluted medium from treated cells by human IL-23 ELISA kit (Invitrogen, 88-7237-88) with 2× dilution or Duo ELISA kits (R&D technologies) for human CXCL10 (DY266) with 10–20× dilution, IL-17A (DY317) with 2× dilution, TNF-α (DY210) with 5–20× dilution, IFN-γ (DY285B) with 2× dilution and IL-10 (DY217B) with 2× dilution according to the manufacturer’s instructions, and with the manufacturer’s standard curve ranges and limits of detection. Values below detection were input as zero. Absorbance was analyzed on a Synergy HTX S1LFA reader (BioTek). GainData^®^ (36) was used with a 4-parameter logistic regression standard curve for the calculation of cytokine concentrations. For cytokine analysis from fibroblast and keratinocyte cell media, Bio-Plex Pro Human Cytokine 8-plex Assay (Bio-Rad, M50000007A) was used, and run on a Bio-Plex Multiplex immunoassay system (Bio-Rad, BioPlex^®^ 200). The media samples were analyzed non-diluted except for analysis of IL-8 (with 20× dilution), and quantified against standard curves according to manufacturer’s instructions, and with the manufacturer’s standard curve ranges and limits of detection. Values below detection were input as zero.

### 2.6 Quantitative real-time PCR (qrt-PCR)

RNA from immune cells was isolated by the Quick-RNA Miniprep Kit (Zymo Research, R1054) and RNA concentration measured by 260/280 absorbance on a Synergy HTX S1LFA plate reader (BioTek). cDNA was synthesized by iScript cDNA kit (BioRad, 1708890) and run with the PrimePCR™ Probe Assays (BioRad) for human and the SsoAdvanced™ Universal Probes Supermix (BioRad, 1725280) for 40 cycles (activation: 2:00 at 95 °C, dissociation: 0:10 at 95 °C, annealing and amplification: 0:45 at 60 °C) on a CFX96 Real-Time PCR System (BioRad). The mean of HPRT and B2M Cts was used for normalization. Relative mRNA levels were transformed into linear by the 2^–ΔΔ*Ct*^ method. Fold changes of RNA expression (RQ) relative to unstimulated controls were used for statistical analysis. PrimePCR™ Probe Assays were: qHsaCEP0040184 (TNF), qHsaCEP0053880 (CXCL10), qHsaCEP0054112 (IFNB1), qHsaCJP0034602 (IL23), qHsaCIP0030549 (HPRT1) and qHsaCIP0029872 (B2M).

RNA from fibroblasts and keratinocytes was isolated by the RNeasy Midi Kit (#75144, Qiagen, Germany) according to the manufacturer’s instructions. RNA concentration was measured with a Nanodrop One instrument (Thermo Scientific) with 340 nm baseline correction. cDNA was generated from 1 μg total RNA using Taqman Reverse Transcription Reagents (#N8080234, Thermo Fisher Scientific, MA, USA) with random hexamers according to the manufacturer’s protocol for mono-cultured cells, and with LunaScript RT SuperMix kit (E3010, New England Biolabs) for co-cultured cells. RT-qPCR analysis was carried out using TaqMan Gene expression Master Mix (#4369510, Life Technologies, Thermo Fisher Scientific) for mono-cultured cells, and with Luna Universal Probe qPCR MasterMix (M3004, New England Biolabs) for co-cultured cells. All RT-qPCR analysis were performed on QuantStudio5 (Applied Biosystems, Foster City, CA, USA) PCR System. The amplification using TaqMan Master Mix was initiated at 50 °C for 2 min, followed by denaturation at 95 °C for 10 min, then 40 cycles of denaturation at 95 °C for 15 s followed by annealing of TaqMan probes and amplification at 60 °C for 1 min. The amplification using Luna Universal Probe Master Mix was run with Fast cycling profile for 43 cycles (activation: 1:00 at 95 °C, dissociation: 0:15 at 95 °C, annealing, and amplification: 0:35 at 60 °C). EEF1A1 was used for normalization, and the relative gene expression (RQ) was calculated against the unstimulated control by the comparative 2^–ΔΔ*Ct*^ method. RT-qPCR analysis was performed on 3 biological replicates, each with 3 technical replicates. All commercial TaqMan^®^ primers (Thermofisher) and probes were: Hs00265885_g1 (EEF1A1), Hs00230071_m1 (NFKBIZ) and Hs00161488_m1 (S100A7).

### 2.7 Western blot

Cells were washed twice with cold PBS before lysis in Pierce™ RIPA buffer (89900, Thermo Scientific) containing Protease and Phosphatase inhibitor (A32959, Thermo Scientific) for 30 min on ice. The lysates were centrifuged at 13.000 rpm for 20 min at 4 °C. Protein concentrations were determined using DC Protein Assay (BioRad). 20 μg protein was loaded per well of SDS-PAGE NuPage 12% Bis-Tris gels (NP0342BOX, Invitrogen), and gels were run in 3-(N-morpholino) propane sulfonic acid (MOPS) SDS running buffer (NP0001, Invitrogen) at 200 V for approximately 50 min. Following electrophoresis, the proteins were transferred onto nitrocellulose membranes (iBlot3 Transfer stacks, IB33001, Invitrogen) using an iBlot Gel Transfer Device (Invitrogen). All membranes were blocked with 2% ECL Prime blocking agent (RPN418V, Cytiva) in TBS-tween for 1 h at room temperature (RT). Primary and secondary antibodies were diluted in 0.2% blocking agent and incubated for 1.5 h at RT (or overnight at 4 °C) with gentle shaking. Membranes were washed 3 × 10 min with TBS-tween after both incubations. ECL plex™ Rainbow™ Fluorescent marker from Cytiva (#RPN850E, MA, USA) was used as a molecular weight marker. Proteins were scanned and visualized using G: BOX Chemi XX6/XX9-(Syngene, India). Primary antibodies used were: mouse anti-α-tubulin (1:10.000, #T5168, Sigma Aldrich, USA), mouse anti-Psoriasin (1:1000, NB100-56559, Novus Biological). Secondary antibody used were: ECL Plex goat-a-mouse IgG Cy3 (cat.no PA43009, Cytiva).

### 2.8 Statistical analysis

Statistical analyses were performed in GraphPad Prism 10.0.1. Data of primary immune cells was analyzed by non-parametric Friedmans test and all treatments were compared to each other by Dunn’s test. Data from immune cells without any stimuli or treatment (none) are shown for illustration and were not included in statistical comparisons for better clarity. All other data was analyzed by ANOVA and all treatments were compared to each other. Shown are boxplots from min. to max. showing all points or bar graphs for means with standard deviation. *P*-values of <0.05 were considered statistically significant and only statistically significant comparisons are shown.

## 3 Results

### 3.1 HRO is well tolerated and taken up by cells

As a first step to characterize effects of HRO at cellular level, the range of tolerable supplementation was analyzed. The immune cells were treated with HRO for 24 h and skin cells were treated for 96 h, according to the further treatment set-up. Up to 500 μg/mL HRO were tolerated in the immune cells and 50 μg/mL HRO in the skin cells without impairment of cellular viability (Supplementary Figure 2). Cellular uptake of fatty acids from HRO has been described by us before, reporting significant increases in the n3-PUFAs EPA and DHA in the here used MDMs and keratinocyte/fibroblast co-culture (30).

### 3.2 HRO supplementation limits secretion of IL-23 from macrophages

**FIGURE 1:**
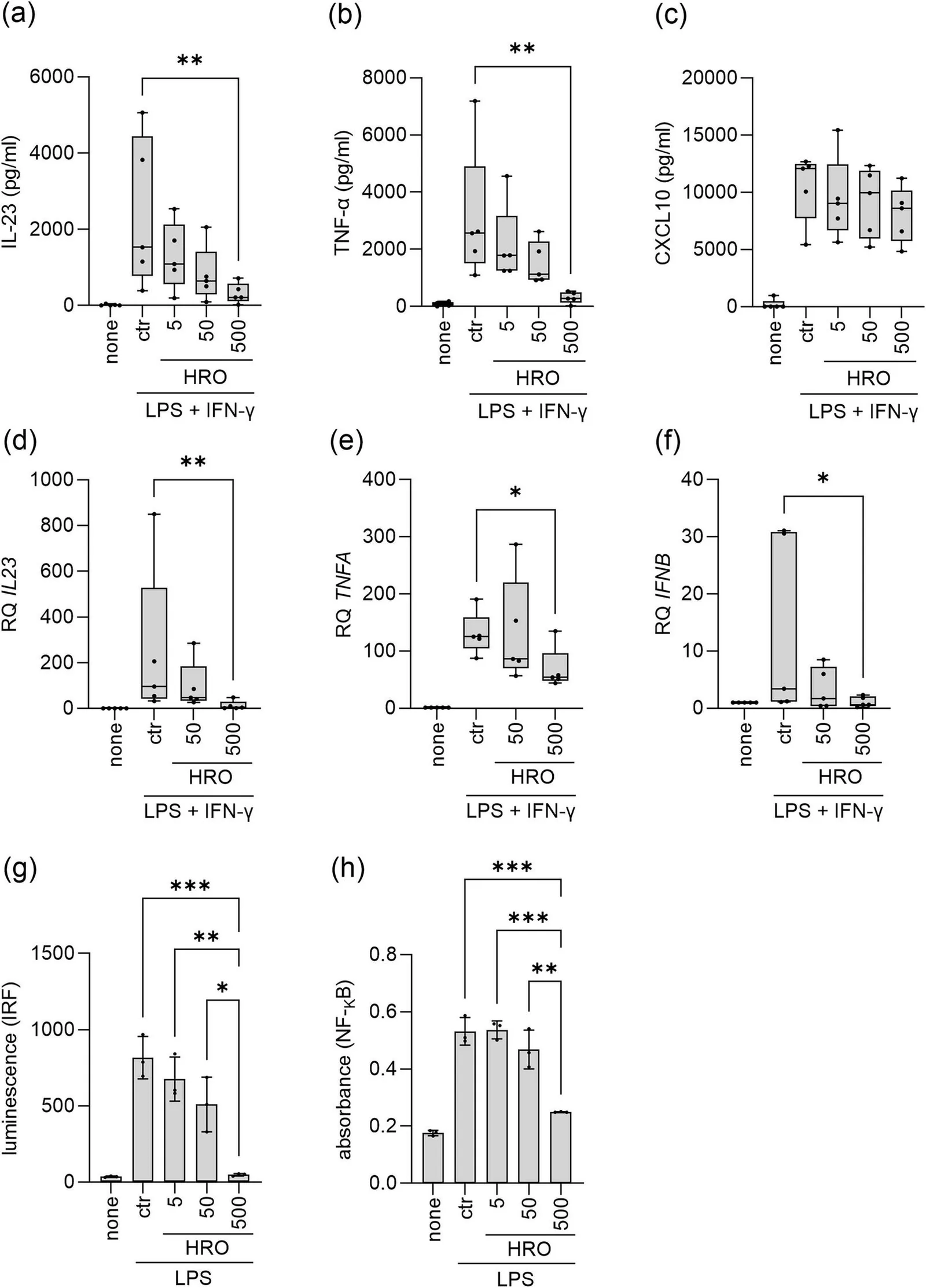
Effects of herring roe oil (HRO) on macrophages. Human primary monocyte-derived macrophages (MDMs) were pretreated with HRO at indicated concentrations (5–500 μg/mL) for 16 h, followed by stimulation with LPS (1 μg/mL) and IFN-γ (50 ng/mL) for 24 h and analysis of secreted IL-23 **(a)**, TNF-α **(b)** and CXCL10 **(c)** by ELISA (*n* = 5). Or followed by stimulation with LPS and IFN-γ for 4 h for assessment of mRNA levels by qrt-PCR for IL23 **(d)**, TNFA **(e)**, and IFNB **(f)** (*n* = 5). Reporter activity of NF-κB **(g)** and IRF **(h)** reporters in THP-1 macrophages treated with HRO as indicated (5–500 μg/ml) for 16 h, followed by stimulation with 10 ng/ml LPS for 24 h (*n* = 3). All treatments were compared to each other. *P ≤ 0.05; **P ≤ 0.01; ***P ≤ 0.001.

On the cellular level, the development and progression of psoriasis is driven by the reciprocal over-activation of immune and skin cells (37). Macrophages and DCs in the skin produce, among other cytokines, IL-23 as an important driver of the inflammatory cycle in psoriasis (38). Macrophages are potential targets for the treatment of psoriasis (39), and were selected for the evaluation of HRO effects, due to their more consistent induction of IL-23 (data not shown) and previously described use as models for IL-23 signaling (34). MDMs were pretreated by HRO overnight, followed by stimulation with LPS and IFN-γ. Secretion of IL-23 and TNF-α were significantly reduced by 500 μg/mL HRO (Figures 1a–c). On mRNA level, the expression of *IL23* was similarly reduced after pretreatment with 500 μg/mL HRO, as well as the expression of *TNFA* and *IFNB* as respective targets of NF-κB or IRF (Figures 1d–f). In a set-up with short inflammatory stimulation (2 h), HRO was effective when THP-1 cells were pretreated with HRO for 16 h, but not when pretreated for 2 h or HRO added at inflammation induction (Supplementary Figure 3). Finally, we observed a strong and dose-dependent reduction of both NF-κB and IRF activity in THP-1 reporter cells, suggesting that dampening effects of HRO occur upstream of the activation of these transcription factors (Figures 1g, h).

### 3.3 HRO supplementation limits IL-17 secretion from T-cells

**FIGURE 2:**
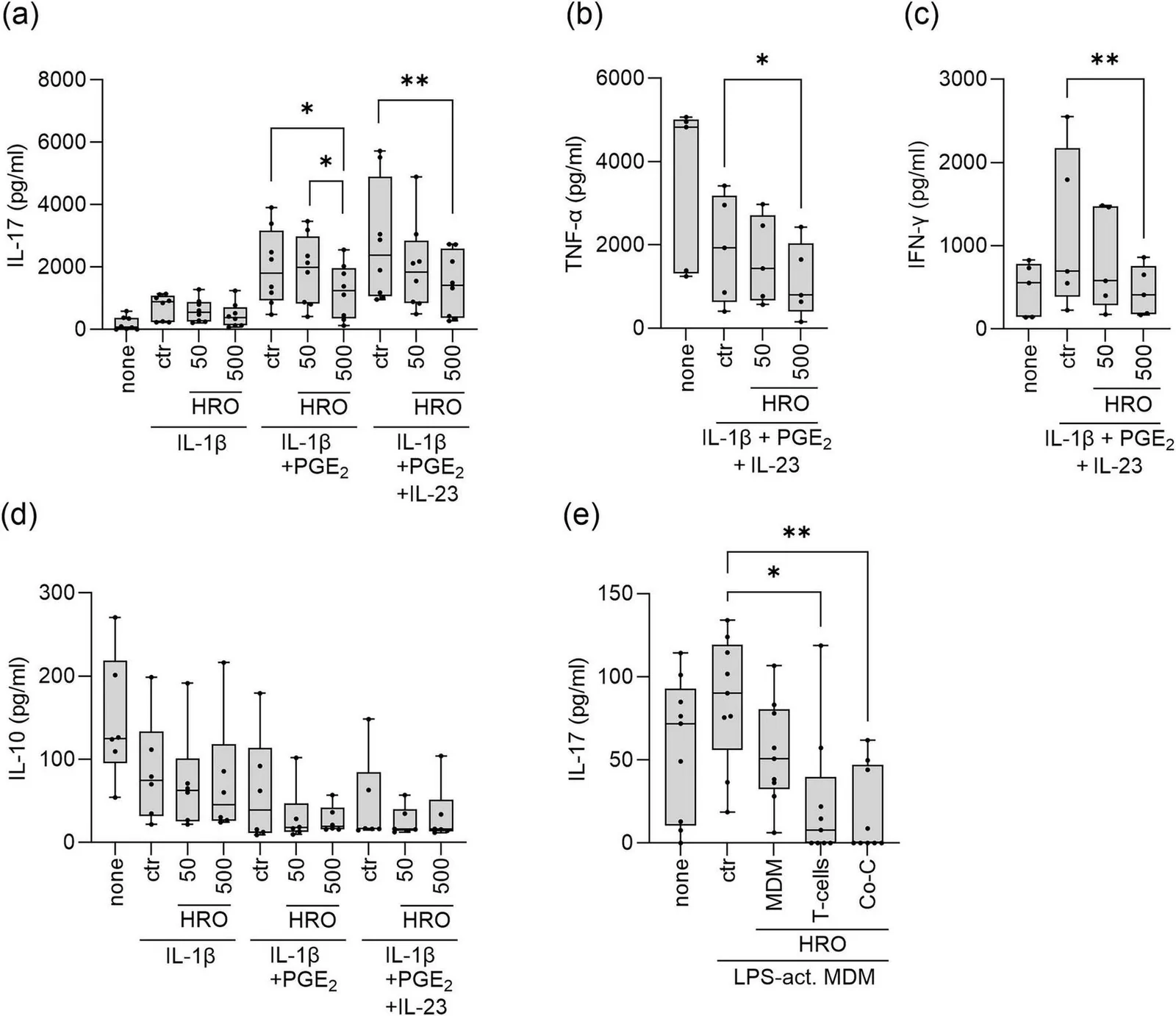
Effects of herring roe oil (HRO) on T-cells. IL-17 **(a)**, TNF-α **(b)**, IFN-y **(c)** and IL-10 **(d)** secretion assessed by ELISA from activated T-cells stimulated with indicated combinations of IL-1β (20 ng/ml), PGE_2_ (5 μM) and IL-23 (25 ng/ml) in presence or absence of 50 or 500 μg/ml HRO for 3 days (*n* ≥ 5). **(e)** IL-17 secretion assessed by ELISA from co-cultures of activated monocyte-derived macrophages (MDMs) and autologous T-cells. Indicated cell types were treated by HRO (50 μg/ml). MDMs were pretreated by HRO for 16 h, then activated (LPS-act. MDM) by LPS (1 μg/ml) for 24 h, followed by addition of T-cells. T-cells were pretreated with HRO for 16 h before addition to MDMs. Addition of HRO upon initiation of the co-culture is indicated as Co-C (*n* = 9). Treatments were compared to each other for each stimuli combination. *P ≤ 0.05; **P ≤ 0.01.

Increased levels of IL-23 lead to the differentiation of naive T-helper-cells into Th17-cells, which secrete high amounts of IL-17 as a pathologic driver in psoriasis (2). T-cells were treated with combinations of IL-1β, PGE_2_ and IL-23 for 3 days to induce secretion of IL-17 (40). Co-treatment with HRO did significantly reduce IL-17 secretion for all inducing stimulations (Figure 2a). TNF-α was not induced by stimulation but still reduced by HRO (Figure 2b). IFN-γ was induced by stimulation and reduced by HRO, suggesting that also Th1-cells might be affected by HRO (Figure 2c). IL-10 was suppressed by stimulation with PGE_2_ as described before (40) and not further changed by HRO (Figure 2d). IL-17 secretion was also induced by co-culture of T-cells with activated MDMs as described before (34). IL-17 secretion in this model was significantly reduced when either T-cells or the co-culture was supplemented with HRO (Figure 2e), while pretreatment of MDMs was not effective.

### 3.4 HRO reduces the expression of NFKBIZ and S100A7, and proliferation of co-cultured keratinocytes and fibroblasts

**FIGURE 3:**
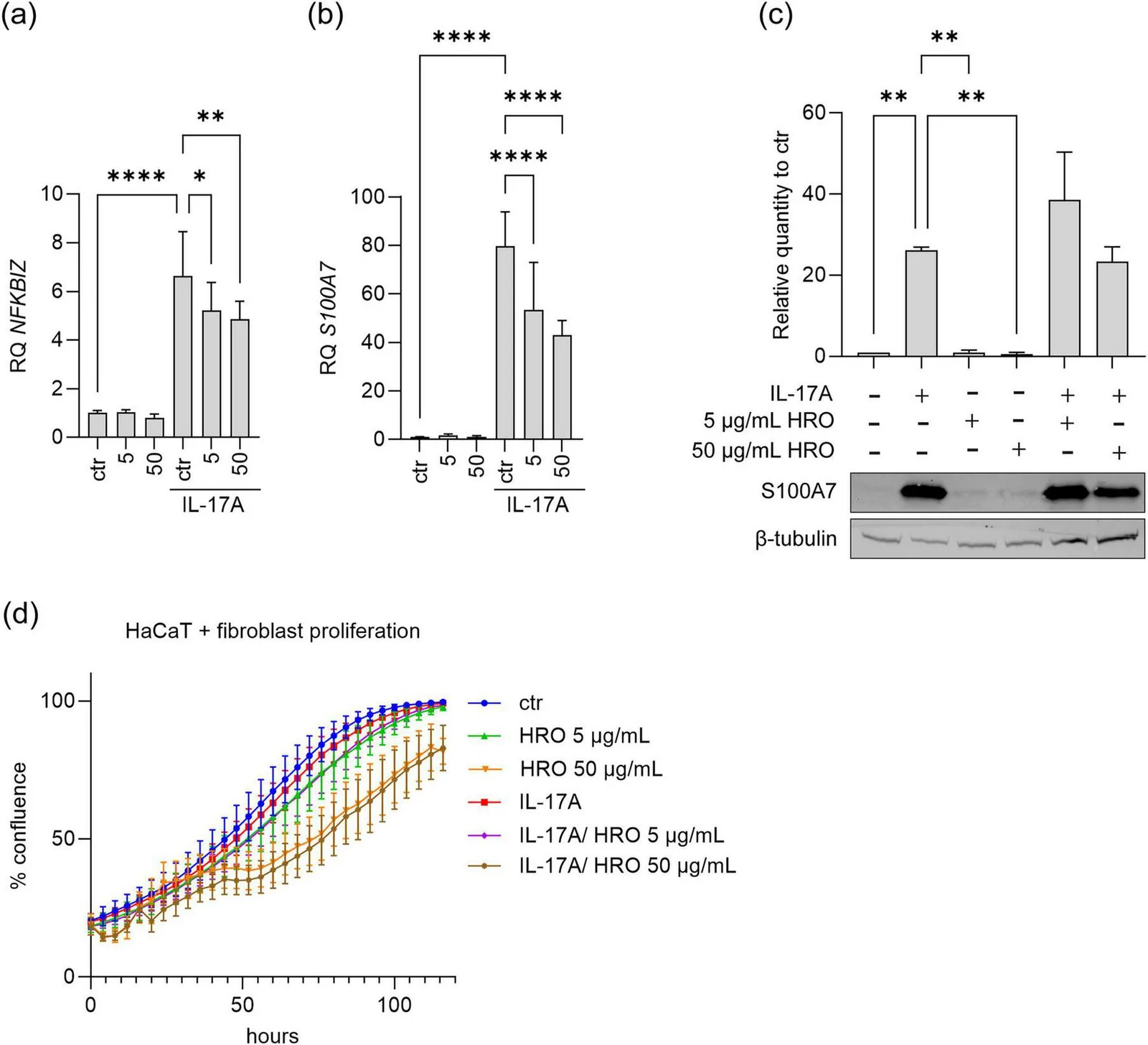
Effects of herring roe oil (HRO) on skin cells. RT-qPCR analysis of the psoriasis-related response genes *NFKBIZ* **(a)** and *S100A7* **(b)** (*n* = 3), and Western blot analysis of the S100A7 protein **(c)** (*n* = 3) on RNA and protein isolated from a physical co-culture of keratinocytes and fibroblasts. Keratinocytes were co-cultured with fibroblasts for 5 days before stimulation with 100 ng/mL IL-17A, and/or 5 or 50 μg/mL HRO for 4 days. For proliferation analysis keratinocytes and fibroblasts were co-cultured in a 5:1 ratio for 1 day before 100 ng/mL IL-17A, and/or 5 or 50 μg/mL HRO was added **(d)**. Monitoring of cell confluence was started immediately after addition of stimuli and subsequently monitored every 4 h for 120 h. The proliferation curve shown is a representative of 4 graphs, each with 4 technical replicates. Western blot analysis was performed on 3 biological replicates, each with 1 technical replicate. A representative picture is shown. All treatments were compared to each other. For RT-qPCR: only statistical significance to control (ctr) with IL-17A stimuli are shown. *P ≤ 0.05; **P ≤ 0.01; ****P ≤ 0.0001.

IL-17 activates keratinocytes in the psoriatic skin, increasing their cytokine secretion, and interfering with proliferation and differentiation (6). IL-17 also induces *NFKBIZ*, that is a positive regulator of NF-κB and a central mediator of IL-17-driven effects (10). HRO at both 5 and 50 μg/ml significantly reduced the IL-17-induced expression of *NFKBIZ* and its downstream target, the psoriasis-associated gene *S100A7* in a physical co-culture of keratinocytes and fibroblasts (Figures 3a, b). At the protein level, the reduction in S100A7 was non-significant across biological replicates [Figure 3c, Supplementary Figure 4 (full membranes)]. We further analyzed the proliferation of physically co-cultured keratinocytes and fibroblasts that was not significantly affected by neither IL-17 nor 5 μg/mL HRO, or their combination. However, with 50 μg/mL HRO, we observed a significant reduction in proliferation after 70 h, both with and without IL-17 (Figure 3d). Investigations of keratinocytes and fibroblasts when co-cultured in a TW-system, or monocultured for proliferation studies, revealed that both cell types reduced the IL-17-induced expression of *NFKBIZ* with 5 μg/mL HRO (Supplementary Figures 5a, d). Similar to the observations in the physical co-culture, the IL-17-induced expression of *S100A7* was reduced by the addition of 5 μg/mL HRO in keratinocytes, and proliferation was reduced by addition of 50 μg/mL HRO, regardless of IL-17 treatment (Supplementary Figures 5b, c). However, in fibroblasts, 5 μg/mL HRO significantly enhanced both the IL-17-mediated expression of *S100A7*, and significantly increased proliferation after 30 h, regardless of IL-17 stimulation (Supplementary Figures 5e, f). These data identify some opposite effects of HRO on the studied skin cells, where, *S100A7* expression and proliferation were reduced in keratinocytes, while increased in fibroblasts. Supplementation with 50 μg/mL HRO resulted in cloudy cell media, over estimating the confluence measurements of both cell types (data not shown). This effect was visually reduced over time by keratinocytes, removing the overestimation of confluence at later time-points in the growth curve, whereas this turbidity sustained in the fibroblast monoculture. This observation explains the bumpy 50 μg/mL HRO graphs in early time-points in Figure 3d, Supplementary Figure 5c as well as the high confluence graphs for treatment of fibroblasts with 50 μg/mL HRO in Supplementary Figure 5f. We also wanted to evaluate potential effects of HRO on the inflammatory signaling especially from keratinocytes in the skin cell co-culture. Thus, we have analyzed IL-17-induced cytokines secreted by keratinocytes in the TW co-culture with fibroblasts but could not observe effects of HRO in this setting (Supplementary Figure 6).

## 4 Discussion

Psoriasis has a complex pathophysiology including the inflammatory interaction of many skin and immune cell types (41). While there is a plethora of systemic treatments indicated for patients with moderate-to-severe psoriasis, there are limited treatment options for mild-to moderate psoriasis. Patients with non-severe psoriasis may not have marked systemic inflammation, and immunosuppressive treatments with their side-effects may not be appropriate, thus only topical treatment or phototherapy are available. HRO has previously shown beneficial effects on clinical signs in psoriasis (27, 28), and decreased activation of neutral killer cells and monocytes systemically (29). Here, we have characterized effects of HRO on models of skin and immune cells present in psoriatic skin and demonstrated immunomodulatory effects on macrophages and T-cells and anti-proliferative effects on keratinocytes, which are relevant in the pathophysiology of psoriasis. HRO decreased the secretion of IL-23 and TNF-α from MDMs, further in line with the decreased expression of these cytokines at the mRNA level and limited activation of NF-κB and IRF in a reporter cell line. The impact of HRO on these central inflammatory pathways suggests that also other downstream cytokines might be reduced by HRO. Meta-analyses of clinical trials with n-3 PUFAs for the treatment of psoriasis have not found a strong causal relationship (22), which may be due to differences in the chemical nature of the studied lipid formulations, such as lipid classes, the detailed composition of fatty acids or the content of precursors for SPM formation (23–26). On a molecular level n-3 PUFAs are well known to exert anti-inflammatory effects via multiple pathways (20) that should be further investigated for HRO. These include activation of the Free fatty acid receptor 4 (FFAR4)/G-protein coupled receptor 120 (GPR120), the nuclear receptor Peroxisome proliferator-activated receptor gamma (PPAR-γ), activation of the antioxidative response element Nuclear factor erythroid 2-related factor 2 (NFE2L2), or induction of miRNAs, among many others (42). MDMs were pre-treated with HRO before addition of inflammatory stimuli, as we have seen that in the case of short inflammatory stimulation (2 h) as used for the expression analysis of cytokine mRNA, HRO was only effective when pre-treated for longer times. This suggests that time for intracellular signaling or metabolism is needed for HRO to exert its observed anti-inflammatory effect. We have previously shown that HRO-supplemented MDMs in this set-up produce SPMs (30) and this SPM profile is likely to be specific for HRO as different marine oils have previously been shown to lead to the production of different SPMs (26). SPMs have established resolving actions (19), and a causative connection to the here presented results should be investigated in future studies, for example by inhibition of SPM metabolizing enzymes. Likewise, there are several effects on psoriatic signaling described for the SPMs that are also produced by HRO-treated MDMs. Resolvin D1 (RvD1) was the most important contributor to changes in SPM levels from the HRO-treated MDMs (30) and has been shown by others to limit NF-κB activation and cytokines of the IL23/IL17 axis (43). RvE3 was also produced by HRO-treated MDMs and reduced IL-23 release from macrophages in another study (44). HRO supplementation also limited IL-17 and IFN-γ secretion from T-cells, thus markedly interfering with inflammatory T-cell signaling. Also, pretreatment of T-cells with HRO led to reduced IL-17 secretion when induced by co-culture with MDMs. The ineffective HRO pretreatment of MDMs in this case might have been due to the sequence of the experiment, where HRO treatment of T-cells or the co-culture was done more shortly before analysis. This *in vitro* set-up also has limitations, as the chosen supplementation regime for HRO could not be sustained for long enough for analysis of surface markers and transcription factors in T-cells and should be optimized further, especially as n-3 PUFAs as well as RvD1 have previously been shown to also reduce Th17 differentiation in other models (45, 46). Hence, HRO interferes with the inflammatory circle in psoriasis by dampening both IL-23 and TNF-α secretion from macrophages and IL-17 secretion from T-cells. These are the main cytokines also targeted by biologics for the treatment of more severe psoriasis, underlining the relevance of HRO-mediated effects in the context of psoriasis (1, 17). It would of course also be interesting to analyze HRO effects on IL-23 secretion from DCs, as this cannot be estimated from our results. However, the generation of monocyte-derived DCs involves incubation with IL-4, which abrogates IL-23 signaling (47) and also myeloid derived DCs have shown limited IL-23 secretion without further modification of the signaling pathways (48). In keratinocytes, we did not observe effects of HRO on IL-17-induced cytokines as measured by us and it has to be noted that Il-17 is an important, but not the only pro-inflammatory stimuli in psoriatic skin. One challenge of this study was to find relevant supplementation regimes for the different assays. In MDMs, cytokine expression and secretion are quickly induced upon inflammatory stimulation, and we have seen that HRO should be present for a certain time to be effective. Also, in this system, it was possible to show that HRO can be effective when pre-treated but removed during inflammatory stimulation. On the other hand, more time was needed to allow for activation of T-cells or cell-cell communication of co-cultures, where it seemed more relevant for HRO to be present simultaneously. Future direct comparative studies would be needed to determine if HRO acts differently on different inflammatory signaling pathways. It is important to keep in mind that *in vitro* models cannot reflect the complexity or timeline *in vivo*, where it took several months before clinical effects were seen (27, 28), but allow the investigation of local effects that cannot be measured systemically. HRO did reduce the IL-17-induced expression of *NFKBIZ* and its downstream target *S100A7* in keratinocytes, thus showing immunomodulatory effects. The *NFKBIZ*-encoded protein IκBζ is an important driver of IL-17 mediated psoriatic effects and an interesting therapeutic target (9, 10). The role of S100 proteins in psoriasis is complex and context dependent, and a broader literature indicates that S100 proteins exert diverse functions such as intracellular Ca2+ sensing, response to extracellular stimuli, induction of cytokines, and energy metabolism depending on the cellular context (12). In the fibroblasts, *NFKBIZ* was reduced while contradictory *S100A7* was increased by HRO, showing a different effect on *S100A7* in fibroblasts compared to keratinocytes. Interestingly, a recent *in vivo* study investigating IL-23 inhibition, revealed dynamic fibroblast states changing between the onset and resolution of chronic inflammation (16). The non-significant effect of HRO on S100A7 protein levels measured in the protein lysates from direct co-culture containing protein from both keratinocytes and fibroblasts, supports the opposite *S100A7* expression pattern observed in these two cell types. We also observed a dampening effect of HRO on keratinocyte proliferation that could correspond to the reduction of scaling in psoriatic skin in a clinical trial with HRO (28). The anti-proliferative effect of HRO was observed independently of IL-17, which might indicate a restoring activity of HRO in case of inflammatory challenge, but also a regulatory role in normal conditions. In some contexts, S100A7 promotes proliferation (12) similar to our data, where a reduction in *S100A7* expression by HRO coincides with reduced proliferation of keratinocytes. In fibroblasts on the other hand, stimulation *in vitro* with recombinant S100A7 has an anti-proliferative and anti-fibrotic effect (49). The signaling around S100 proteins in psoriasis is complex and effects are highly context-dependent, thus further studies are needed to clarify the molecular mechanisms behind the effect of HRO on fibroblasts in our study. It would be interesting to focus on the composition of the extracellular matrix, which is changed in fibrotic conditions but also in psoriatic conditions (50). Fibrotic processes are inflammation-driven (51). In this context, it could also be interesting to investigate the potential effect of HRO on mesenchymal stem cells and their exosomes, which are another emerging and promising treatment approach for psoriasis as well as other wound healing models (52, 53). In a psoriatic skin model, the n-3 PUFAs DHA, EPA and alpha-linoleic acid (ALA) had a reducing effect on keratinocyte proliferation and DHA and ALA also normalized differentiation (54–56). In addition, we have previously shown that keratinocyte/fibroblast co-cultures produce SPMs upon supplementation with HRO (30) and one of the SPMs observed by us, Protectin D1 (PD1), did reduce skin thickness and scaling in a mouse model of psoriasis (57). It could be further interesting to study the direct effect of the produced SPMs on keratinocyte proliferation and differentiation. Overall, here we have focused on some major aspects of psoriasis pathophysiology and, as HRO has shown effects on all here tested cell types, HRO might exert even further effects on the numerous cell types and pathways involved in psoriasis.

In conclusion, HRO displays anti-inflammatory effects on macrophages and T-cells relevant for resolving the inflammatory component of psoriatic disease through limiting secretion of IL-23 and IL-17, respectively. Immunomodulation by reduction of IL-17-induced *NFKBIZ* and *S100A7*, along with reduced proliferation of keratinocytes, supports an effect of HRO on skin cells with relevance in psoriatic pathophysiology.

The observed effects in this study show that HRO might act on distinct levels on the IL-23/IL-17 axis, where HRO may affect several cell types simultaneously. Recent data from our lab demonstrate that the uptake of HRO in these cell types promotes broad lipid mediator biosynthesis (30). This, taken together with effects on cellular responses in both immune cells and skin cells shown here, may further indicate that HRO can impact intracellular processes relevant in psoriasis. This gives promise for HRO as a potential oral treatment modality for psoriatic disease, especially as safe long-term use of HRO has previously been shown in a clinical trial (27, 28). To develop HRO as a treatment option for patients with non-severe psoriasis, the systemic cellular and clinical effects must be further studied through a clinical program with extended clinical trials. The clear dampening effect on inflammation in immune cells could also be relevant in other IMIDs and warrants further investigation.

## Data Availability

The original contributions presented in this study are included in this article/Supplementary material, further inquiries can be directed to the corresponding author.
