# Supplementary material for: Herring roe oil exerts immunomodulatory effects on the IL-17/23 signaling axis in immune and skin cells as models for psoriasis
Source: Front Med (Lausanne). 2026 Aug 24;13:1684328. doi: 10.3389/fmed.2026.1684328 (PMC13548772; doi:10.3389/fmed.2026.1684328)
Supplement: Supplementary file 1 [file Data_Sheet_1.docx]

Supplementary Figures

**Fig. S1. Example of T-cell purity analysis and gating strategy.** T-cell purity was checked by staining for CD4, CD3 and CD14, analyzed by Flow cytometry and visualized in FCSalyzer 0.9.22-alpha, and was routinely above 95 %.

**Fig. S2. Viability.** Viability after treatment with indicated concentrations of herring roe oil (HRO) (5 - 5000 μg/ml) for 24 h in human primary monocyte-derived macrophages (MDMs) (a), THP-1 macrophages (b) and human primary CD4^+^ T-cells (c), and after treatment with HRO (5 and 50 μg/ml) for 96 h in HaCat keratinocytes (d) and human primary fibroblasts (e). Shown are relative viability (%) as compared to untreated control indicated as dashed line (100 %). All treatments were compared to the untreated control. * = P ≤ 0.05; *** = P ≤ 0.001; **** = P ≤ 0.0001)

**Fig. S3. Timeseries of herring roe oil (HRO) treatment.** THP-1 macrophages were treated with 50 μg/ml HRO. The time of HRO addition (-16 h, -2 h) is indicated as time before stimulation with 10 ng/ml LPS for 2 h. Shown are relative mRNA expression (RQ) as assessed by qrt-PCR for TNFA (a), or IFNB (b) and IL23 (c). All treatments were compared to each other. * = P ≤ 0.05; ** = P ≤ 0.01

(a)

(b)

**Fig. S4. Full membranes**. Shown are the full membranes for S100A7 (a) and β-tubulin in the lowest band (b), corresponding to the cropped bands shown in Figure 3. The membrane for S100A7 was divided into three parts before staining.

**Fig. S5. Effects of herring roe oil (HRO) on keratinocytes and fibroblasts.** RT-qPCR analysis of the effect of 5 µg/ml HRO on the expression of psoriasis related response genes *NFKBIZ* (a) and *S100A7* (b) in co-cultured keratinocytes, and in co-cultured fibroblasts (d) and (e) on RNA isolated from a Transwell co-culture of keratinocytes and fibroblasts. Cell confluency of mono-cultured keratinocytes (c) and mono-cultured fibroblasts (f) plated 1 day prior to addition of 100 ng/mL IL-17A, and/or 5 or 50 µg/mL HRO. Monitoring of cell confluence was started immediately after addition of stimuli and subsequently monitored every 4 hours for 120 hours. RT-qPCR analysis was performed on 3 biological replicates, each with 3 technical replicates. The proliferation curves shown are representatives of 4 graphs each, each performed with 4 technical replicates. All treatments were compared to each other. Only statistically significant comparisons are shown. **** = P ≤ 0.0001.

**Fig. S6. Effects of herring roe oil (HRO) on skin cell cytokines.** IL-8 (a), IFNγ (b), IL-4 (c), IL-6 (d), TNFα (e) and GM-CSF (f) secretion assessed by BioPlex ELISA on cell media from keratinocytes co-cultured with fibroblasts in Transwells after 4 days stimulation with 100 ng/mL IL-17A and/or 5µg/mL HRO. All treatments were compared to each other. Only statistically significant comparisons are shown. * = ≤ 0.05; ** = P ≤ 0.01; *** = P ≤ 0.001; **** = P ≤ 0.0001.
